# Targeted pharmacotherapy after somatic cancer mutation screening

**DOI:** 10.12688/f1000research.9040.2

**Published:** 2016-09-20

**Authors:** Thomas M. Polasek, Karen Ambler, Hamish S. Scott, Michael J. Sorich, Peter A. Kaub, Andrew Rowland, Michael D. Wiese, Ganessan Kichenadasse

**Affiliations:** 1 Department of Clinical Pharmacology, Flinders Medical Centre and Flinders University, Adelaide, Australia; 2 Department of Genetics and Molecular Pathology, SA Pathology, Adelaide, Australia; 3 School of Pharmacy and Medical Science, University of South Australia, Adelaide, Australia; 4 Department of Medical Oncology, Flinders Centre for Innovation in Cancer, Adelaide, Australia

**Keywords:** : targeted pharmacotherapy, oncology, precision medicine, dabrafenib erlotinib, bevacizumab, malignant melanoma, non-small cell lung cancer, metastatic colorectal cancer

## Abstract

Many patients with solid tumours are treated with targeted pharmacotherapy based on the results of genetic testing (‘precision medicine’). This study investigated the use of targeted drugs after OncoFOCUS™+ *KIT* screening in patients with malignant melanoma, non-small cell lung cancer and metastatic colorectal cancer, and then audited the results against the National Comprehensive Cancer Network (NCCN) guidelines. Patients who were not indicated for targeted pharmacotherapy did not receive such treatment (99%, 100/101). Of the patients indicated for targeted drugs, 79% (33/42) received treatment according to NCCN guidelines. In 48% (20/42) of these patients the results from OncoFOCUS™+ *KIT* screening were required for targeted drug selection, with the remaining 52% (22/42) prescribed drugs independent of the screening results for various reasons. This study highlights the growing importance of precision medicine approaches in directing pharmacotherapy in medical oncology.

## Introduction

Over the last 20 years the molecular profiles of many solid tumours have been characterised. The discovery of specific variants in critical proteins that influence cancer pathogenesis has seen the development of ‘targeted pharmacotherapy’ – drugs that selectively inhibit unique molecular targets in tumour cells. Compared to traditional cytotoxic agents, targeted drugs have considerable benefits in the treatment of cancer, including improved response rates and less toxicity ^1^.

This field of cancer therapeutics is rapidly evolving with several hundred ongoing clinical trials. However, there are no local guidelines in Australia to inform the prescribing of targeted pharmacotherapy. As a consequence, clinicians often use resources from pharmaceutical companies, conference presentations, journal publications or recommendations from other countries, such as the US National Comprehensive Cancer Network (NCCN) guidelines, for their clinical practice. Although the NCCN guidelines are not always directly applicable for practice in Australia, these are reviewed annually, are freely available ( www.nccn.org), and have best practice recommendations for targeted pharmacotherapy use in selected cancers.

**Figure 1.**
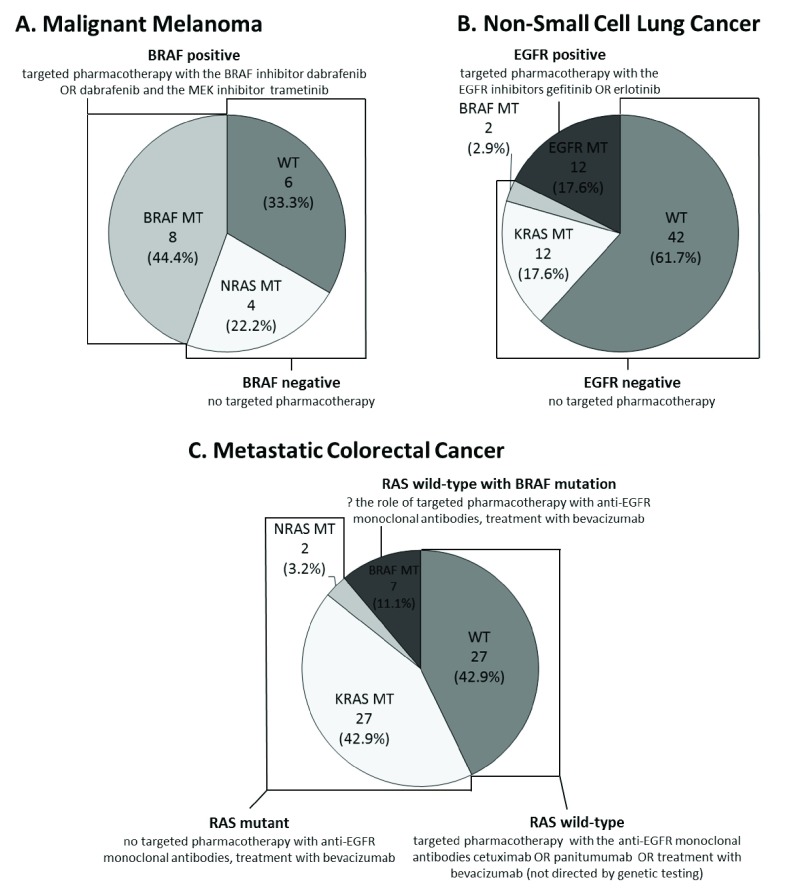
OncoFOCUS™+ *KIT* results, molecular cancer classifications, and the 2015 NCCN guideline recommendations for targeted pharmacotherapy.

In addition to the well documented role of estrogen/progesterone receptor and HER-2 testing in selecting therapies for breast cancer, three other important cancers in Australia, malignant melanoma, non-small cell lung cancer (NSCLC) and metastatic colorectal cancer (mCRC), now have targeted drugs available for treatment based on genetic testing. Dabrafenib, with or without trametinib, is used for malignant melanoma with activating *BRAF* mutations (‘BRAF positive’) ^2^, whereas imatinib can be used for *KIT*-mutated melanoma. Patients with NSCLC that harbours activating *EGFR* mutations (‘EGFR positive’) are recommended the EGFR inhibitors erlotinib or gefitinib ^3^. Two monoclonal antibodies that also inhibit EGFR (cetuximab and panitumumab) significantly improve survival in patients with mCRC that is *RAS* wild-type (WT), whereas those with mutations in *RAS* are essentially insensitive ^4^. Bevacizumab is a selective inhibitor of VEGR that is also used in mCRC but response rates are independent of *RAS* status i.e., genetic testing is often not necessary for treatment decisions. Bevacizumab is frequently used first-line in combination with chemotherapy regimens such as FOLFOX, FOLFIRI and CapeOX ^5^. Figure 1 shows the 2015 NCCN recommendations for targeted pharmacotherapy based on the molecular profiles of the cancers investigated in this study ^6– 9^.

OncoFOCUS™+ *KIT* is a somatic cancer mutation screen offered by SA Pathology ( www.sapathology.sa.gov.au) for clinicians in South Australia. The test analyses the oncogenes *KRAS*, *NRAS*, *EGFR*, *BRAF* and *KIT*. Clinically significant mutations in these genes are reported as either ‘no mutation detected’ (WT) or as a specific mutation e.g., *BRAF* V600E. Screening with OncoFOCUS™+ *KIT* has recently been introduced at the Flinders Centre for Innovation in Cancer (FCIC), an academic healthcare centre located in the southern suburbs of Adelaide that specialises in research and treatment of cancer. Given this introduction into clinical practice, and the lack of local prescribing guidelines, the aim of this study was to audit targeted pharmacotherapy use after screening against the latest NCCN recommendations.

## Methods

A retrospective chart-based audit of OncoFOCUS™+ *KIT* results and targeted pharmacotherapy use was conducted. Ethics approval for the study was granted by the Southern Adelaide Human Research Ethics Committee (application 137.15). Inclusion criteria were: ≥ 18 years, diagnosis of malignant melanoma, advanced NSCLC or mCRC, record of attendance at the FCIC in 2014, and OncoFOCUS™+ *KIT* results reported in 2014. The electronic patient system OACIS was searched for genetic test results and relevant discharge summaries, multi-disciplinary team meeting summaries and electronic and/or hardcopy case notes were reviewed to determine pharmacotherapy use. In a small number of cases (21), information about medications used in private practice was confirmed with the treating oncologist. Retrieval of data was conducted over a 3 month period between June–August 2015. Results were presented as descriptive data or as a percentage.

## Results

Sixty percent (90/149) of the cohort were male and 40% (59/149) were female, with a mean average age of 67.6 years (range 34 to 91 years). At the audit cut-off date, 48.3% (72) were alive, 49.7% (74) were deceased and the living status of 2.0% (3) could not be determined. There were similar numbers of patients with NSCLC (68) and mCRC (63) but a smaller number of patients with malignant melanoma (18).

OncoFOCUS™+ *KIT* results for patients with malignant melanoma, NSCLC and mCRC are shown in Figures 1A–C, respectively. All patients were *KIT* WT. Importantly, the cohort had similar cancer mutation rates as previously reported. Forty four percent with malignant melanoma had an activating *BRAF* mutation (40–60% reported ^10^), 17.6% had *EGFR*-positive NSCLC (10–20% reported ^11^), and 46% had *RAS* mutant mCRC (40% reported ^12^). These data suggest that the FCIC cohort is representative of the wider population.

**Figure 2.**
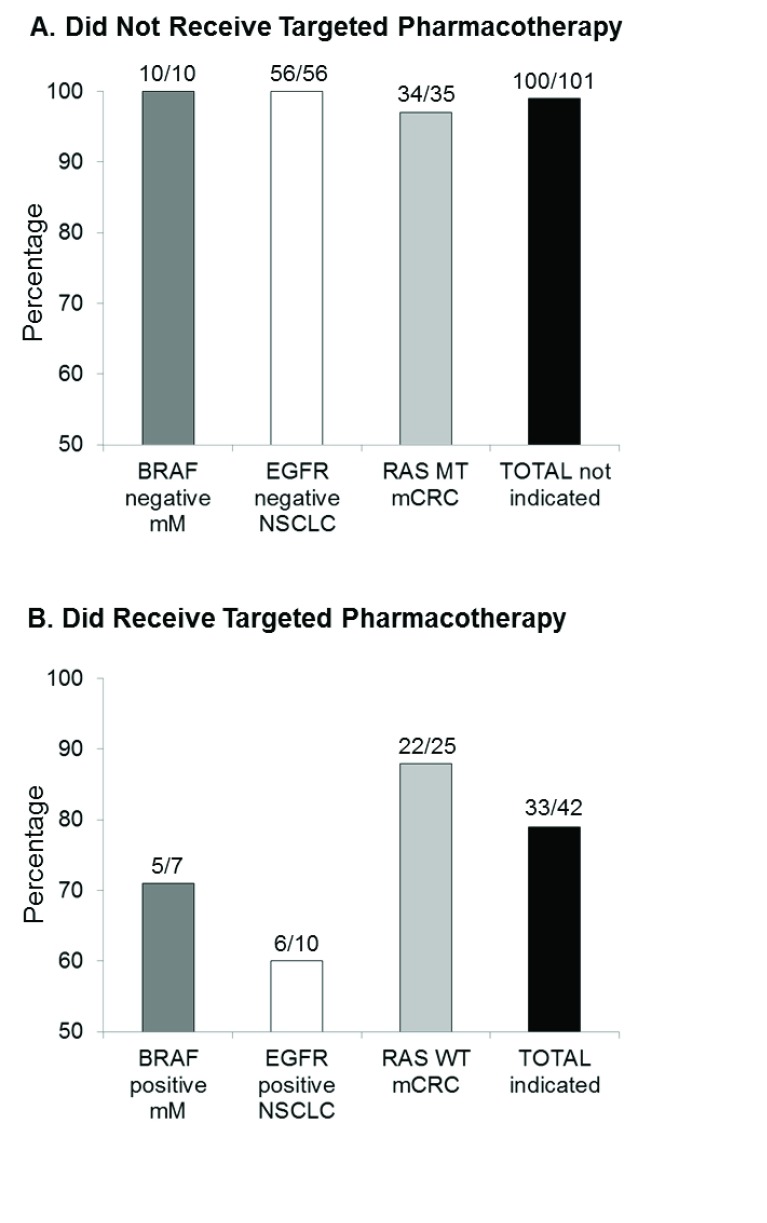
Percentage of patients who ( **A**) did not receive targeted pharmacotherapy or ( **B**) did receive targeted pharmacotherapy according to NCCN guidelines after OncoFOCUS™+ *KIT* screening.

Of the 149 patients included, only 6 patients (3.8%) were excluded from the analysis of targeted pharmacotherapy use due to incomplete records. Figure 2 shows the percentage of patients who received or did not receive a targeted drug according to NCCN guidelines. Appropriately, almost all patients not indicated for targeted pharmacotherapy did not receive targeted pharmacotherapy (99%, 100/101). Of the 42 patients in the total cohort indicated for targeted therapy, 79% (33/42) received such treatment according to NCCN guidelines ( Figure 2). Of the 25 patients with mCRC that was *RAS* WT, 36% (9/25) had targeted pharmacotherapy directed by OncoFOCUS™+ *KIT* with an anti-EGFR drug (8 cetuximab, 1 panitumumab), 52% (13/25) received bevacizumab, and 12% (3/25) did not receive a targeted drug in contrast to NCCN guidelines. If bevacizumab in *RAS* WT mCRC is excluded, 48% (20/42) of the total indicated cohort received appropriate targeted drugs following OncoFOCUS™+ *KIT* screening i.e., required genetic test results for a targeted drug to be prescribed.

OncoFOCUS screening raw data Frequency of oncogene mutations and targeted pharmacotherapy in malignant melanoma, advanced non-small cell lung cancer, and metastatic colorectal cancer. Click here for additional data file. Copyright: © 2016 Polasek TM et al. 2016 Data associated with the article are available under the terms of the Creative Commons Zero "No rights reserved" data waiver (CC0 1.0 Public domain dedication).

## Discussion

This study is the first to report utilisation rates of targeted pharmacotherapy after OncoFOCUS™+ *KIT* screening. As expected, patients who were not indicated for targeted pharmacotherapy did not receive such treatment. In contrast, the use of targeted drugs directed by OncoFOCUS™+ *KIT* screening was relatively low (48%).

This result may be explained by factors that are independent of OncoFOCUS™+ *KIT* results. First, the use of bevacizumab in mCRC does not require genetic testing – it is considered equivalent to cetuximab and panitmumumab in *RAS* WT mCRC and was given first-line to most patients with mCRC at FCIC ^6^. This is confusing because bevacizumab is a targeted drug by definition, selectively inhibiting VEGR. Second, targeted drugs for NSCLC and mCRC were subsidised by the Australian Pharmaceutical Benefits Scheme (PBS) in 2014 as second-line only. Thus, patients on first-line chemotherapy appropriately did not receive targeted drugs, despite having mutations suggesting they may benefit from such treatment. During 2014, anti-EGFR drugs became indicated for first-line treatment of *EGFR*-positive NSCLC and were funded by the PBS ^13^. Likewise, cetuximab and panitumumab are now PBS-subsidised as first-line treatment in *RAS* WT mCRC ^13^. Not differentiating between first- and second-line targeted pharmacotherapy is a major limitation of the study (note that half the cohort was still alive at the audit cut-off date, precluding a more complete analysis of the temporal relationships between screening and targeted pharmacotherapy use). Third, a number of patients had genetic testing close to the end of life. These patients were considered too unwell for further oncology treatment, or declined targeted drugs when offered, preferring to transfer to palliative care.

The exact role of targeted drugs for some of the cancer mutations reported by OncoFOCUS™+ *KIT* is unclear. For example, approximately 5–9% of colorectal cancers (7.9% in this study) are characterised by a specific mutation in the *BRAF* gene (V600E) which causes constitutive activity, in theory bypassing inhibition by cetuximab and panitumumab and potentially making them insensitive ^14^. In the colon cancer NCCN guidelines, *BRAF* mutation testing is currently optional and not part of decision making for anti-EGFR drugs ^6^. A recent meta-analysis suggests that there is currently insufficient evidence to conclude that patients with mCRC harbouring *BRAF* mutations should be denied anti-EGFR therapy over concerns of poor efficacy ^15^. However, there are conflicting views on whether BRAF status should influence use of anti-EGFR therapy ^16, 17^, and hence some clinicians may potentially utilise BRAF status to make treatment decisions. This highlights the difficulty of auditing medical oncology prescribing where guidelines and the underlying evidence are rapidly evolving.

The OncoFOCUS™+ *KIT* screening panel is currently limited to five oncogenes. The status of other oncogenes that may influence treatment decisions is determined separately. For example, patients with NSCLC are also tested for *ALK* rearrangements, and if positive are eligible for treatment with crizotinib (although it is not currently PBS-subsidised for this indication) ^8^. Once the importance of emerging genetic alternations is established in these cancers, such as *MET* amplifications, *ROS1* and *RET* rearrangements, and *HER2* mutations, the OncoFOCUS™+ *KIT* screening panel could be expanded to facilitate more complete molecular diagnosis.

In conclusion, this study showed that most patients at the FCIC receive pharmacotherapy for their cancer according to NCCN guidelines (93%), and that the results of a somatic cancer mutation screening test are applied reasonably well to drug selection. Precision medicine approaches are of increasing importance when directing pharmacotherapy in medical oncology.

## Data availability

The data referenced by this article are under copyright with the following copyright statement: Copyright: © 2016 Polasek TM et al.

Data associated with the article are available under the terms of the Creative Commons Zero "No rights reserved" data waiver (CC0 1.0 Public domain dedication). 

F1000Research: Dataset 1. OncoFOCUS screening raw data, 10.5256/f1000research.9040.d127508 ^18^
