# Supplementary material for: Targeted pharmacotherapy after somatic cancer mutation screening
Source: F1000Res. 2016 Sep 20;5:1551. Originally published 2016 Jun 30. [Version 2] doi: 10.12688/f1000research.9040.2 (PMC5247780; doi:10.12688/f1000research.9040.2)
Supplement: Peer review history

# Peer review: Referee response for version 2

**DOI:** 10.5256/f1000research.10374.r19477

**Published:** 2017-01-19

**Authors:** Nimit Singhal

**Affiliations:** 1 Cancer Centre, Royal Adelaide Hospital, Adelaide, SA, Australia

The article was updated and edited as per suggestions of primary reviewer. The updated article has appropriate abstract, content and conclusions. The graphs are easy to understand and clear.

I have read this submission. I believe that I have an appropriate level of expertise to confirm that it is of an acceptable scientific standard.

# Peer review: Referee response for version 2

**DOI:** 10.5256/f1000research.10374.r16463

**Published:** 2016-09-21

**Authors:** Andrew A. Somogyi

**Affiliations:** 1 Discipline of Pharmacology, School of Medicine, Faculty of Health Sciences, University of Adelaide, Adelaide, SA, Australia

I have read this submission. I believe that I have an appropriate level of expertise to confirm that it is of an acceptable scientific standard.

# Peer review: Referee response for version 1

**DOI:** 10.5256/f1000research.9728.r15849

**Published:** 2016-08-24

**Authors:** Andrew A. Somogyi

**Affiliations:** 1 Discipline of Pharmacology, School of Medicine, Faculty of Health Sciences, University of Adelaide, Adelaide, SA, Australia

The title suitably addresses the content of the article; in the Abstract, after the second last line, a comment should be made regarding the 52% who were screened by the test but did not receive the targeted drugs. The content of the article in general is of a high standard, it could be improved to the casual and inexpert reader by clarifying where KIT, KRAS and NRAS fit within the overall picture as BRAF deals with MM, EGFR with NSCLC and RAS with mCRC; and of the 6 exclusions the number in each cohort (targeted versus no targeted) could be stated. Finally, Figure 2 is unclear and a disconnect between the figure (BRAF, EGFR, RAS positive, negative) and the legend (…received or did not receive targeted pharmacotherapy), to include actual numbers and not just percentages would also make it clearer. The conclusions are most appropriate and it succinctly highlights the complex interplay between testing, prescribing, guidelines, evidence and funding all of which are rapidly changing but are not connected, all of which have universal implications.

I have read this submission. I believe that I have an appropriate level of expertise to confirm that it is of an acceptable scientific standard.

# Response

**Published:** 2016-09-12

**Authors:** Thomas Polasek

**Affiliations:** Flinders University Medicine, Australia

We thank Prof Somogyi for these very useful comments and suggestions regarding our manuscript. We have now uploaded version 2 to incorporate changes based on the review as described below:

1. *‘after the second last line, a comment should be made regarding the 52% who were screened by the test but did not receive the targeted drugs’. *We have now added the following in the abstract, ‘with the remaining 52% (22/42) prescribed drugs independent of the screening results for various reasons.’
2. *‘by clarifying where KIT, KRAS and NRAS fit within the overall picture’.* In the introduction we have now added that KIT testing is used for selecting imatinib for metastatic melanoma e.g. ‘whereas imatinib can be used for *KIT*-mutated melanoma’. We have not added further comments about the differences between *KRAS* and *NRAS*, since they are considered together in targeted drug selection for mCRC (as already described in the manuscript by referring to *RAS*), and mutations in these for metastatic melanoma and NSCLC are not currently indications for targeted drug selection.
3. *‘and of the 6 exclusions the number in each cohort (targeted versus no targeted) could be stated’.* We have already stated that these 6 could not be included in the analysis due to incomplete records (Results first line paragraph 3).
4. *‘Figure 2 is unclear and a disconnect between the figure (BRAF, EGFR, RAS positive, negative) and the legend (…received or did not receive targeted pharmacotherapy), to include actual numbers and not just percentages would also make it clearer’.* We have added a new Figure 2 to make the distinction clearer, including raw data.
